# Proteomic Signature of Extracellular Vesicles Associated with Colorectal Cancer

**DOI:** 10.3390/molecules28104227

**Published:** 2023-05-22

**Authors:** Natalia Soloveva, Svetlana Novikova, Tatiana Farafonova, Olga Tikhonova, Victor Zgoda

**Affiliations:** Orekhovich Institute of Biomedical Chemistry, Pogodinskaya 10, 119121 Moscow, Russia; novikova@ibmc.msk.ru (S.N.); farafonova.tatiana@ibmc.msk.ru (T.F.); ovt@ibmh.msk.su (O.T.)

**Keywords:** extracellular vesicles, proteomic signature, mass spectrometry, colorectal cancer, SRM, stable-isotope-labeled peptide standards

## Abstract

The proteins of extracellular vesicles (EVs) provide proteomic signatures that reflect molecular features of EV-producing cells, including cancer cells. Detection of cancer cell EV proteins is of great interest due to the development of novel predictive diagnostic approaches. Using targeted mass spectrometry with stable-isotope-labeled peptide standards (SIS), we measured in this study the levels of 34 EV-associated proteins in vesicles and whole lysate derived from the colorectal cancer (CRC) cell lines Caco-2, HT29 and HCT116. We also evaluated the abundance of 13 EV-associated proteins (FN1, TLN1, ITGB3, HSPA8, TUBA4A, CD9, CD63, HSPG2, ITGB1, GNAI2, TSG101, PACSIN2, and CDC42) in EVs isolated from blood plasma samples from 11 CRC patients and 20 healthy volunteers. Downregulation of TLN1, ITGB3, and TUBA4A with simultaneous upregulation of HSPG2 protein were observed in cancer samples compared to healthy controls. The proteomic cargo of the EVs associated with CRC represents a promising source of potential prognostic markers.

## 1. Introduction

Colorectal cancer (CRC) is a disease characterized by uncontrolled division of abnormal cells in the colon or rectum epithelium. In 2020, according to the World Health Organization, CRC was ranked second (916,000 cases) and third (1.93 million cases) in terms of mortality and prevalence of cancer, respectively (https://www.who.int/ru/news-room/fact-sheets/detail/cancer (accessed on 19 May 2023)). This undoubtedly characterizes CRC as a socially significant disease. The risk of the disease increases with lifestyle habits, e.g., smoking, alcohol consumption, and frequent consumption of red and processed meat [1]. In addition, according to the literature, visceral obesity is associated with unfavorable prognosis in men.

CRC is a heterogeneous disease. In 70% of cases, CRC occurs sporadically, and 10–30% is associated with a family predisposition [2,3]. At the same time, 5–7% of patients with a family predisposition to CRC had a diagnosis of familial adenomatous polyposis and non-polypoid colorectal cancer (FAP and NNRC). In these cases, the tumors were characterized by marker mutations, e.g., *APC*, *hMSH2*, *MLH1*, *PMS1*, *PMS2*, *MSH6* [4].

The transformation of normal colonic epithelium to a precancerous lesion (adenoma) and ultimately to an invasive carcinoma is associated with the accumulation of genetic mutations. According to the CRC development theory, the clonal evolution of mutations provides advantage in survival and proliferation of cells with following mutation accumulation. Thus, this vicious cycle of CRC progression leads to further invasion and metastasis [5]. Generally, the occurrence of CRC is associated with chromosomal and microsatellite instability of the genome and epigenetic regulation through DNA hypermethylation [6]. Approximately 70–85% of cases (including FAP) of CRC development through the chromosomal instability associated with mutations in the *APC* gene or loss of the long arm of chromosome 5q (which harbors the *APC* gene), KRAS oncogene mutations, loss of the long arm of chromosome 18q (genes *DCC*, *SMAD2* and *SMAD4*) and deletion in the short arm of chromosome 17p, which contains the tumor suppressor gene *TP53* [7]. For other cases, including NNRK, a high level of microsatellite instability is typical. Examples of such instability are mutations in the genes *MLH1*, *MSH2*, *MSH6,* and *PMS2*, the protein products of which are responsible for proper DNA mismatch repair [8]. In addition, such epigenetic regulation as CpG-hypermethylation of DNA and activation or suppression of *BRAF* and *MLH1* genes are important in the carcinogenesis of CRC [9].

Unfortunately, CRC is usually detected at late stages, which leads to unfavorable prognosis. Early diagnosis of CRC, however, improves the outcome of the disease [10]. The five-year survival rate is 90% for CRC diagnosed at an early stage compared with just 13% for those diagnosed at late stages (https://www.cancer.net/cancer-types/colorectal-cancer/statistics (accessed on 19 May 2023)).

Intensive screening programs for CRC have been implemented worldwide. These programs are based on immunochemical analysis of stool for hemoglobin, guaiac analysis of stool for occult blood, and finger rectal examination [11]. However, endoscopy (colonoscopy, rectoromanoscopy, and rectoscopy) remains the basis for CRC diagnosis. Although colonoscopy has proven to be most informative in detecting CRC and adenomatous polyps, this procedure is associated with a risk of serious complications, such as bleeding and perforation of the intestine (0.98% and 0.08%, respectively) [12]. In this context, minimally invasive diagnostic methods, for example, the analysis of tumor markers in the blood plasma, are of practical interest [13,14].

An important prognostic marker for CRC is the level of carcinoembryonic antigen (CEA), which is FDA-approved for clinical application. However, according to the latest data, a weak correlation has been found between CEA levels and tumor size or stage [15]. Therefore, the further search for markers for low-invasion diagnosis and prognosis of this cancer is an urgent task today.

In recent years, interest in liquid biopsy has been growing due to the low invasiveness of this approach. Liquid biopsy is based on the detection of tumor components in body fluids such as blood, urine, etc. [16]. Tumor secretes circulating tumor cells (CTCs), circulating tumor DNA (ctDNA), extracellular vesicles (EVs) and other tumor elements into the extracellular environment. These elements reflect the molecular and cellular landscape of the tumor [17]. EVs have become an area of great interest in liquid biopsy due to their protective double membrane surrounding the contents, such as proteins, DNA and RNA molecules. Furthermore, there are many relatively easy ways to isolate EVs from biological fluids for further analysis [17,18]. Among the EV cargo, proteins are of particular interest because they are responsible for performing essential functions [19,20].

Studies of EV protein composition actively employ shotgun mass spectrometry analysis due to its high sensitivity, selectivity, and high throughput, Shotgun mass spectrometry has enabled the analysis of thousands of proteins in cells, tissues, and fluids [21]. This approach is also used to study EVs as a source of diagnostic markers in CRC [22,23]. In a recent paper, proteins of EVs isolated from CRC cell lines (HCT116, HT29, and SW620) and whole cell lysate proteins were compared using shotgun mass spectrometry proteomics. The comparison showed that proteins involved in CRC signaling pathways, such as *KRAS*, *ARAF*, *mTOR*, *PDPK1*, and *MAPK1*, were found in both cells and EVs. Moreover, EV samples are enriched in *TGFB1* and *TGFBR2* proteins, which play a key role in carcinogenesis of CRC [24].

Targeted mass spectrometry or multiple reaction monitoring (MRM) holds a special place among proteomics methods. As a result of its high sensitivity and selectivity, this method enables the detection of low-abundance proteins, e.g., transcription factors in complex biological matrices, such as blood plasma or cell lysates [25,26]. Another advantage of targeted proteomics is the ability to quantify the desired protein using internal isotopically labeled standards [27]. Moreover, MRM allows for the simultaneous absolute quantitative analysis of numerous proteins, which is of great importance for the quantitative assessment of diagnostically significant proteins [28]. Thus, the MRM technique may be considered an alternative to immunochemical assays, which are conventionally used in clinical diagnostics.

In a recent study utilizing a targeted proteomic approach on EVs isolated from the plasma of CRC patients, increased expression of proteins of the annexin family (A3, A4, and A11), involved in apoptosis, cell division, and ion transport, was found [29]. Using the tandem mass tag (TMT) method, the authors showed that high levels of total and phosphorylated fibronectin 1 (FN1), haptoglobin (HP), S100A9, and fibrinogen α-chain (FGA) in EVs of CRC blood samples were associated with cancer progression. In addition, parallel reaction monitoring (PRM) confirmed that the presence of FGA protein in EVs can help to distinguish colonic adenoma and CRC patients from healthy individuals [22]. Using two-dimensional LC-MS/MS analysis, accurate inclusion mass screening in conjunction with iTRAQ, and targeted proteomics (product ion scanning (PIS) MS/MS mode), a protein diagnostic panel was defined. This panel consists of two plasma EV proteins—CD59 and TSPAN9. Accurate measurements of their content allows the distinguishing of I and II stage patients from healthy donors [30].

Based on the results of our previous studies on the proteome of EVs in a model of CRC cell lines [31], we formed a panel of 34 EV-associated proteins. In this study, using targeted mass spectrometry, the content of 28 proteins was detected in cultural media-derived EVs and whole cell lysate (WhL) originating from CRC cell lines (Caco-2, HT29, and HCT-116). Moreover, 13 of 28 EV-associated proteins were detected and quantified in samples of EVs isolated from blood plasma of CRC patients and healthy volunteers (HV). A proteomic signature consisting of five proteins allowed us to distinguish CRC patients from the control group.

## 2. Results

### 2.1. Targeted Proteomic Analysis of EV-Associated Proteins in HT29, HCT-116, and CaCo-2 Cell Lines

**Figure 1:**
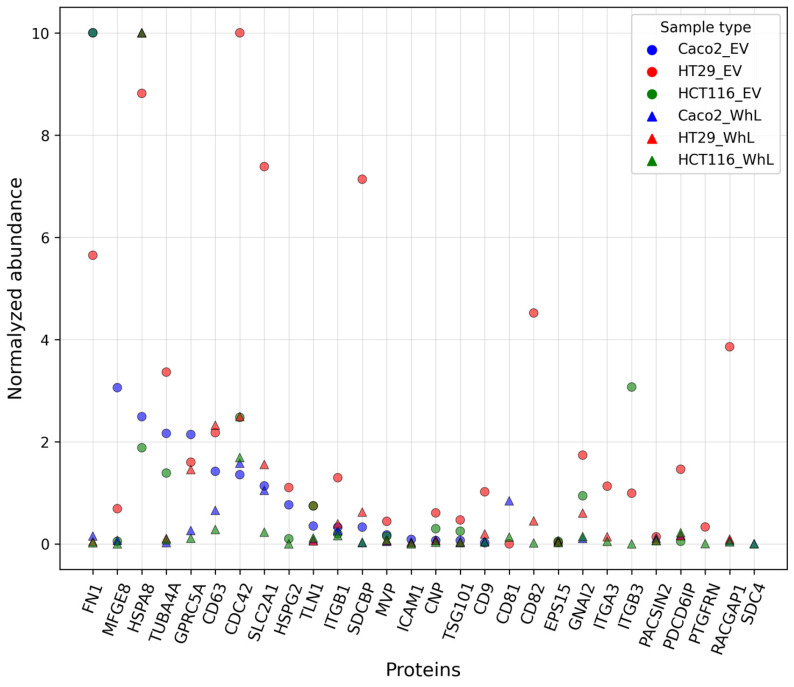
Abundance of 34 peptides uniquely mapped onto 28 EV-associated proteins, which were measured in the extracellular vesicles (EV) and whole cell lysate (WhL) samples derived from CRC HT29, HCT-116, and CaCo-2 cell lines; *Y*-axis is normalized protein abundance.

By applying selected reaction monitoring with stable isotope-labeled peptide standards (SRM/SIS), 34 of the 44 peptides, which were mapped onto 28 EV-associated proteins, were detected and measured in the EV and WhL samples originating from HT29, HCT-116, and CaCo-2 cell lines (Figure 1). Each measurement was carried out in three technical replicates, and the coefficient of variation did not exceed 10% for most of the measurements (68%) (Figure S1). The details on SRM/SIS measurement are provided in Table S1.

Protein abundance was in a range of approximately four orders of magnitude (Figure S2). At the absolute quantification level, the five most abundant proteins, i.e., HSPA8 (104.4 ± 67.3 fmol/µg), CD63 (18.9 ± 8.6 fmol/µg), CDC42 (7.4 ± 2.9 fmol/µg), SLC2A1 (7.4 ± 3.6 fmol/µg), and CD81 (5.6 ± 4.7 fmol/µg), were overrepresented in WhL samples derived from CRC cell lines (Figure S2). The EV-associated proteins with the minimal absolute content, including CD9 (0.05 ± 0.03 fmol/µg), HSPG2 (0.05 ± 0.03 fmol/µg), MFGE8 (0.02 ± 0.01 fmol/µg), EPS15 (0.02 ± 0.01 fmol/µg), and PDCD6IP (0.02 ± 0.02 fmol/µg), were detected in the HCT116-derived EVs.

While basement-membrane-specific heparan sulfate proteoglycan core protein (HSPG2) and integrin beta-3 (ITGB3) (0.49 ± 0.18 fmol/µg) were measured in CRC cell line-derived EV samples, they were not detected in the WhL sample of the respective cell lines.

**Figure 2:**
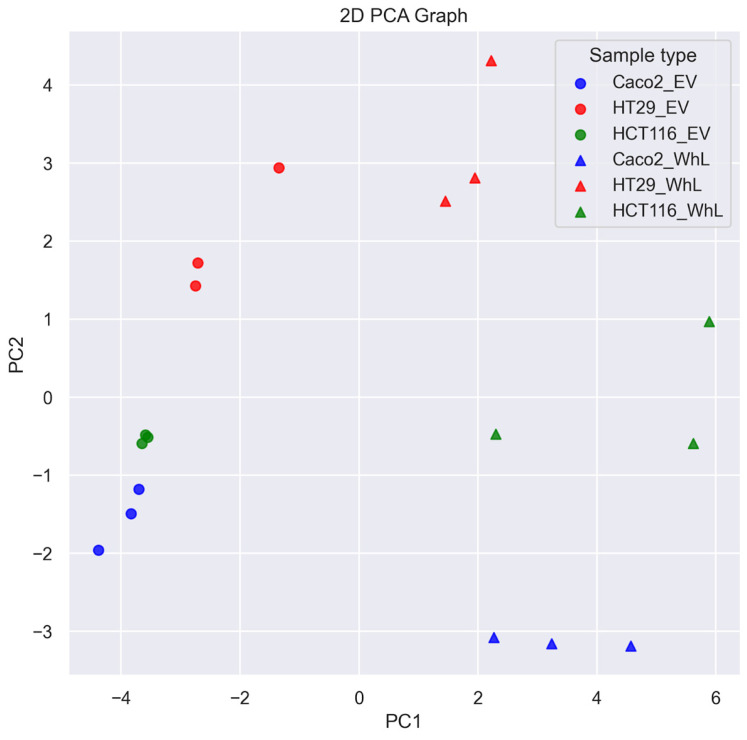
Results of PCA analysis of SRM data obtained for EV and WhL samples derived from CRC cell lines.

The SRM normalized data demonstrate the enrichment of EV samples with almost all EV-associated proteins, especially with FN1, MFGE8, TUBA4A, CDC42, SDCBP, CD9, and CD82 (Figure 1) compared to the respective WhL samples. The results of PCA analysis shown in Figure 2.

The PCA analysis of SRM data demonstrates that the proteomic signature of 28 components distinguishes EV and WhL samples.

### 2.2. Targeted Proteomic Analysis of EV-Associated Proteins in EV Samples Derived from Blood Plasma of CRC Patients and Healthy Volunteers

Applying SRM/SIS, we measured the EV-associated proteins in EV samples derived from blood plasma obtained from 11 CRC patients and 20 HVs. Patient information is shown in Table 1. More detailed information is presented in Table S2. The details on SRM/SIS measurement are provided in Table S1. The coefficient of variation for the most measurements (77%) did not exceed 10% (Figure S3). Fibronectin (FN1) was measured by SRM/SIS with two peptide standards in all the samples studied. The observed contents of two proteotypic peptides, STTPDITGYR and SYTITGLQPGTDYK, for FN1 were correlated, with R^2^ = 0.98 (Figure S4). The calculated average values of the peptide concentrations were considered to be the FN1 abundance in the sample.

**Figure 3:**
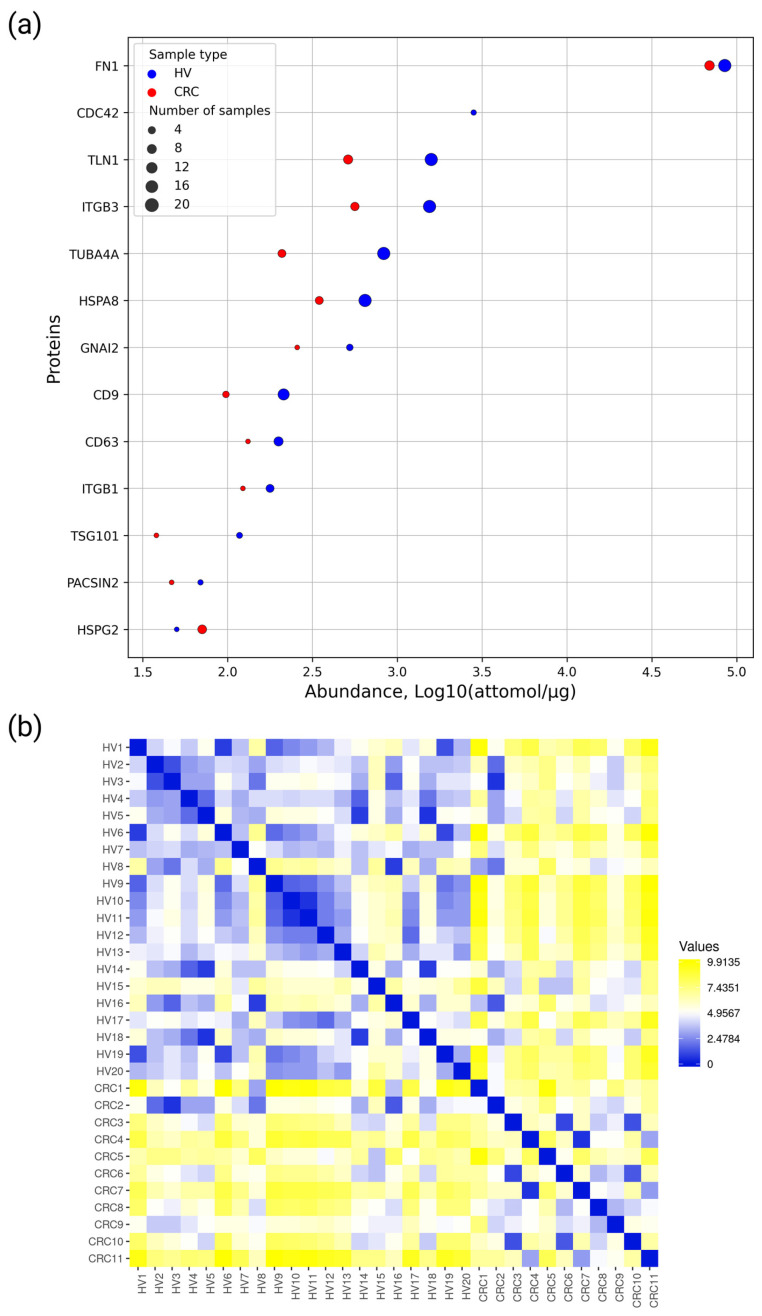
(**a**) The quantitative data on the 13 EV-associated proteins detected in HPL-derived EV samples. Averaged values (log10-transformed attomol/mg of total protein) obtained by MRM/SIS that correspond to healthy volunteers (HVs) and CRC patients (CRC) are shown as blue and red dots, respectively. The size of the markers is proportional to the number of samples in which the proteins were detected. (**b**) Distance matrix of the experimental sample based on SRM/SIS data on 10 EV-associated proteins (FN1, TLN1, ITGB3, HSPA8, TUBA4A, CD9, CD63, HSPG2, ITGB1, and GNAI2). Data were used as log2-transformed fmol/µg. The distance value is color-coded: blue denotes a lower distance and higher similarity between samples.

For EV isolation, we used precipitation with polyglycols (Total Exosome Isolation kit) followed by methanol chloroform protein precipitation. Convenient exosome markers, i.e., HSPA8, CD9, and CD63, were detected at the levels of 0.84 ± 0.69, 0.24 ± 0.12, and 0.2 ± 0.08 fmol/µg, respectively, in HPL-derived EV samples obtained from healthy donors (Figure S5). These markers were also measured at the levels of 0.44 ± 0.19, 0.1 ± 0.03, and 0.15 ± 0.02 fmol/µg, respectively, in HPL-derived EV samples obtained from CRC patients (Figure S5). The SIS/SRM method was used as an alternative to antibody-based approaches, e.g., immunoblot. Overall, as a result of the SRM/SIS analysis, 13 out of 33 targeted proteins were detected in the blood plasma of CRC patients and HVs: fibronectin (FN1), cell division control protein 42 homologue (CDC42), talin-1 (TLN1), integrin beta-3 (ITGB3), tubulin alpha-4A chain (TUBA4A), heat shock cognate 71 kDa protein (HSPA8), integrin beta-3 (ITGB3), guanine nucleotide-binding protein G(i) subunit alpha-2 (GNAI2), tetraspanins CD9 and CD63, integrin beta-3 (ITGB1), tumor susceptibility gene 101 protein (TSG101), and protein kinase C/casein kinase substrate in neurons protein 2 (PACSIN2), and basement-membrane-specific heparan sulfate proteoglycan core protein (HSPG2) (Figure 3). The SRM traces for the detected peptides and the corresponding SIS standards are shown in Figure S4.

Figure 3a demonstrates that the protein abundances were in a range of approximately four orders of magnitude. Ten proteins (FN1, TLN1, ITGB3, HSPA8, TUBA4A, CD9, CD63, HSPG2, ITGB1, and GNAI2) were detected in at least five samples suitable for correlation and distant matrix analysis. Fibronectin was detected in all the samples studied, and it was the most highly abundant at the level of 81.5 ± 23.8 fmol/µg and 74.3 ± 31.2 fmol/µg in EV samples derived from blood plasma of HV and CRC patients, respectively. HSPG2 protein was detected only in one HV EV sample, and in 9 from 11 CRC EV samples at an average level of 0.06 ± 0.03 fmol/µg.

Using SRM/SIS data on 10 EV-associated proteins that were detected in at least five samples, the distance matrix demonstrating the similarity between experimental samples was built (Figure 3b).

The distance matrix demonstrates the similarity of HPL/CRC-derived EV samples. The correlation between samples within each group is moderate.

**Figure 4:**
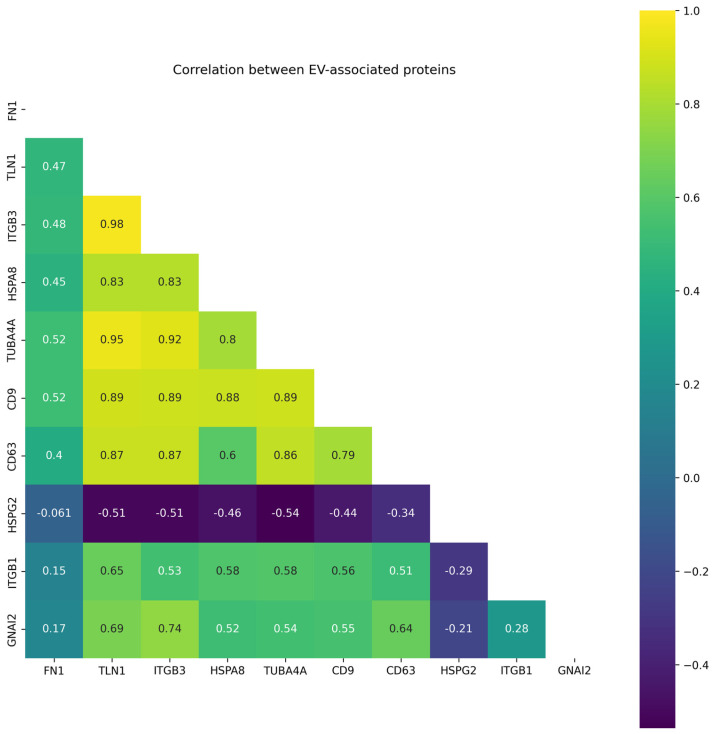
Correlation matrix for 10 proteins detected in at least 5 samples (fmol/µg log2-transformed) of HPL-derived EV samples.

Figure 4 shows correlations of EV-associated protein levels in HPL-derived EV samples. The highest correlation (r > 0.9) was observed for pairs (ITGB3/TLN1, r = 0.98), (TUBA4A/ITGB3, r = 0.92), and (TUBA4A/TLN1, r = 0.95). The levels of CD9 tetraspanin correlate with abundance of ITGB3, TLN1, TUBA4A and HSPA8 proteins (r > 0.85). The content of CD63 correlates with ITGB3, TLN1, TUBA4A levels. The levels of HSPG2 were inversely correlated with the content of other proteins of EV signatures associated with CRC.

**Figure 5:**
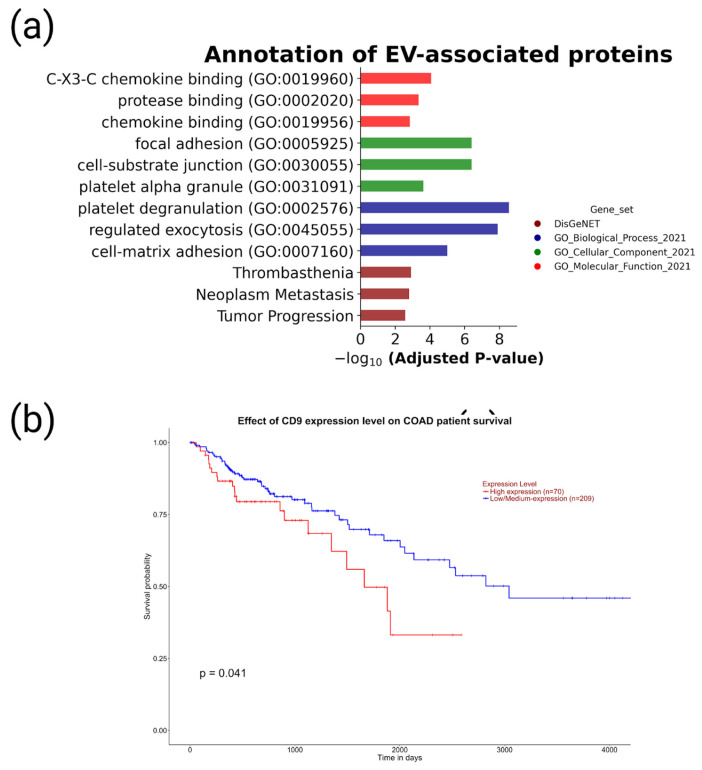
Biological annotation and prognostic value of proteomic signature of EVs associated with CRC. (**a**) Results of overrepresentation analysis of 10 EV-associated proteins (FN1, TLN1, ITGB3, HSPA8, TUBA4A, CD9, CD63, HSPG2, ITGB1, and GNAI2) identified in at least 5 HPL-derived EV samples. Analysis was performed by the Enrichr module of GSEApy bioinformatics tool against libraries based on DisGeNET and GeneOntology data (categories of biological process, cellular components, and molecular function). (**b**) The association of CD9 levels and CRC patient survival calculated in the UALCAN platform. The CD9 transcript expression levels for CRC patients obtained from the Cancer Genome Atlas (TCGA). Survival probabilities associated with high or low/medium CD9 expression.

To elucidate the biological function of the proteomic signature, we performed overrepresentation analysis of 10 EV-associated proteins (FN1, TLN1, ITGB3, HSPA8, TUBA4A, CD9, CD63, HSPG2, ITGB1, and GNAI2) identified in at least 5 HPL-derived EV samples against GeneOntology and DisGeNET databases (Figure 5).

Using the UALCAN online platform, we also checked the predictive potential of EV-associated proteins (Figure 5).

Figure 5a demonstrates that the components of the EV proteomic signature were involved in chemokine and protease binding (GO molecular function). In terms of the GO “cellular components” category, EV proteomic signature components were assigned to focal adhesion, cell–substrate junction, and platelet alpha granule. In terms of the GO Biological process category, EV proteomic signature components were involved in platelet degranulation, regulated exocytosis, and cell-matrix adhesion. According to the DisGeNET database, EV proteomic signature components were associated with thrombasthenia, neoplasm metastasis, and tumor progression. Based on the UALCAN platform data, low expression levels of CD9 were associated with better survival (*p*-value = 0.041).

## 3. Discussion

The molecular composition of EVs is similar to that of the cancer cells that produce them. However, unlike tumor cells that migrate very slowly, vesicles can easily end up in biological fluids such as blood, where they can be detected. Due to these features, the isolation and analysis of the EV molecular cargo, including proteins, fits well into the concept of liquid biopsy [32,33]. Tumor components such as circulating tumor DNA (ctDNA), tumor-associated autoantibodies, and microRNA (miRNA) cells (tumor-educated platelets and circulating tumor cells (CTCs), which are also of interest from a liquid biopsy perspective, are quite unstable and easily degraded by plasma enzymes [34,35]. At the same time, vesicles keep their molecular content secure due to the lipid membrane layer. EVs play a role in many biological processes, including transport of substances and genetic information exchanges [36]. EVs have been established to play a significant role in the creation of the premetastatic environment, and have the ability to promote tumor progression [37]. Additionally, they participate in basic cancer cell processes such as cellular proliferation, tumor expansion, angiogenesis, matrix restructuring, metastasis, and evading immune defenses [38]. Thus, EVs can harbor potential biomarkers both for cancer diagnosis, for predicting outcomes (prognostic markers), and response to treatment (prognostic markers) [39,40].

Using targeted mass spectrometry, we analyzed EV and WhL samples, derived from three CRC cell lines, i.e., Caco-2, HT29, and HCT116. Quantitative data on 28 EV-associated proteins provided molecular patterns, which were called EV proteomic signatures. EV proteomic signatures could distinguish EV and WhL samples, as well as cell lines studied. However, the data on WhL were more scattered within each cell line.

Based on absolute quantitative SRM/SIS data, ITGB3 (HT29- and HCT116-derived EVs) and HSPG2 (all CRC cell line-derived EVs) proteins were detected in the EV samples only compared to respective WhL samples. HSPG2 proteoglycan interacts with extracellular matrix components and is often overexpressed in different types of cancers, including CRC [41,42], that could be associated with tumor growth and invasion. In turn, ITGB3 was shown to promote migration and invasion in CRC, triggered by reactive oxygen species [43]. Considering that EVs carry molecular information to distant organs [37,38], the HSPG2/ITGB3 pair can be involved in prometastatic migration, invasion, and angiogenesis in CRC metastasis.

EV samples can be obtained from blood plasma by different approaches, e.g., ultracentrifugation, ultrafiltration, size-exclusion chromatography, or precipitation with polyglycols [44,45]. The commercially available kits, short and simple protocol, and a low starting sample volume (0.1 mL of HPL/serum) makes the precipitation with polyglycols an attractive strategy for potential EV isolation in clinical settings. In our previous study, we observed that EV precipitation with polyglycols followed by tryptic digestion yielded samples that were incompatible with downstream mass spectrometry [46]. In this study, we added the step of methanol chloroform protein precipitation after EV isolation with a polyglycol-based kit. As a result, we were able to measure EV-associated proteins in EV samples obtained by polyglycol-based precipitation from human blood plasma.

The targeted mass spectrometry analysis resulted in the detection of 13 EV-associated proteins in the HPL-derived EV samples obtained from CRC patients and HVs. Ten of them (FN1, TLN1, ITGB3, HSPA8, TUBA4A, CD9, CD63, HSPG2, ITGB1, and GNAI2) that were measured in at least five samples were denoted as components of an EV proteomic signature associated with CRC. Most of the components were downregulated in HPL/CRC-derived EV samples. Among them, we detected conventional exosome markers, i.e., CD9 and CD63 tetraspanins.

CD9, a member of the tetraspanin superfamily, is a tumor suppressor in many malignancies. A number of studies have linked the increased CD9 expression to a favorable prognosis in patients with CRC [47,48,49]. On the other hand, it was shown that the expression of CD9 was downregulated in most solid tumors, including CRC, and that decreased expression of CD9 strongly correlated with the progression, increased risk of recurrence, angiogenesis, and metastasis [50]. However, according to the UALCAN database, low levels of CD9 are associated with poor outcomes in CRC patients. In our study, low levels of CD9 were identified and compared to healthy controls. Since we used advanced (3 and 4) stage patients in our study, the low levels of this marker may indicate a poor prognosis.

The other member of the tetraspanin superfamily, CD63 protein, is found in endosomes and exosomes. Functionally, it is involved in numerous cancer-associated processes, including cellular differentiation, cell–cell fusion, and cell migration [51]. However, CD63 can have both pro- and anticancer effects, and data on its levels in CRC are controversial. Using immunohistochemistry, it was shown that the increased CD63 expression was associated with a poor prognosis in CRC for a subgroup of patients with metastatic disease. CD63 overexpression was found to be an independent predictor for a negative prognosis for CRC patients at any stage, but especially for patients with metastatic disease. The high expression of tetraspanin is also related to the epithelial-to-mesenchymal transition (EMT) phenotype that occurs in later stages of the disease and is related to worse outcomes for cancer patients [51]. At the same time, in a cohort of patients with CRC, the exosomal marker CD63 expression was lower in tumor tissue compared to adjacent normal mucosa [52].

Cell division control protein 42 homologue (CDC42) has an active oncogenic role in CRC by inhibiting the putative tumor suppressor genes CACNA2D2 and ID4 [53,54]. The high CDC42 expression levels coupled with CACNA2D2 silencing improved the prognosis of the disease. Simultaneous high expression of Cdc42 and silencing of ID4 was found with high incidence (60%) in CRC samples [54]. In this study, we, on the contrary, observed the CDC42 protein decreased levels in HPL-derived EV samples compared to healthy controls. It should be noted that the above studies mainly assessed CDC42 expression at the transcriptome level, and the correlation between protein and mRNA levels could be poor [55,56]. However, CDC42 demonstrates strong involvement in CRC biology, and it was considered a potential therapeutic target for the treatment of the CRC aggressive types [53,54].

Talin1 (TLN1) plays a key role in cancer cell proliferation, adhesion, and migration [57]. TLN1 interacts with integrins, including ITGB3 and ITGB1, which were detected as components of the EV/CRC proteomic signature in this study. Low expression of Talin1 at both the gene and protein level was significantly associated with worse disease-specific survival [57].

Molecular chaperone HSPA8 is involved in the environmental stress response via elimination of misfolded proteins. Compared with normal samples, the expression of HSPA8 increased remarkably in various tumors, including CRC. Moreover, high HSPA8 expression was associated with good prognosis in CRC, as well as in subgroups of male patients with T2–T4 CRC, without lymphatic and perineural invasion. The diagnostic efficacy of HSPA8 in CRC analyzed by ROC gives a reliable value of probability (AUC: 0.889) [58].

Expression of HSPA1B, HSPA4, HSPA5, HSPA6, HSPA8, HSPA9, HSPA13, and HSPA14 was significantly increased, while those of HSPA1A, HSPA2, HSPA7, and HSPA12B were significantly decreased in colon cancer tissues [58].

HSPG2 was the only protein overrepresented in HPL/CRC-derived EV samples compared to healthy controls. As mentioned above, heparan sulfate proteoglycans (HSPG2, a.k.a. perlecan) are complexes of molecules present in the cell membrane and extracellular matrix; they play a vital role in cell adhesion, migration, proliferation, and signaling pathways. Inhibition of HSPG2 leads to significant tumor shrinkage and inhibition of angiogenesis in human CRC tumor xenografts. The proliferation of HCT116 human CRC cells was markedly reduced by inhibition of HSPG2 gene expression by the antisense cDNA, and these effects correlated with a decreased sensitivity to FGF-7x [42]. Moreover, HSPG2 had higher expression in the colon cancer-initiating cell line AG2 compared to HCT116 carcinoma cells. However, it was shown that perlecan gene expression in colon tumor tissue was halved in 12 patients compared to healthy tissue [59].

Individual proteins, such as fibronectin 1 (FN1), have previously been found in cancer-associated vesicles [22]. However, to our knowledge, a simultaneous analysis of 13 proteins identified in this study has not been previously performed [60]. We identified several CRC-associated proteins, e.g., FN1, HSPA8, and TLN1, in the blood plasma obtained from patients with lung cancer. Still, we did not observe CD9, CD63, HSPG2, ITGB1, GNAI2, or CDC42 proteins in those blood plasma samples. To determine the degree of specificity of the CRC/EV signature, additional studies are planned in the future on vesicles associated with other types of cancer, e.g., breast, prostate, and gastric.

Differences in the EV proteomic signatures of healthy volunteers and CRC patients were moderated. Larger cohorts of CRC patients and HVs should be studied. Simultaneous analysis of a panel of protein markers, the so-called proteomic signature, could be more effective than a single analyte.

## 4. Materials and Methods

### 4.1. Cultivation of HT29, HCT-116 and CaCo-2 Cell Lines and EVs Isolation

The CRC cell lines (HT29, HCT-116, and CaCo-2) were obtained from the cell culture bank at the Institute of Biomedical Chemistry (IMBC) in Moscow, Russia. Cells were cultured in a passage number range of 5–10. The cell lines were cultured in a DMEM/F-12 medium without glutamine (PanEco, Moscow, Russia), supplemented with 10% FBS (in the case of the CaCo-2 cell line, FBS content was 20%) (Thermo Fisher Scientific, Waltham, MA, USA), 1% GlutaMAX (Thermo Fisher Scientific, Waltham, MA, USA), 1% non-essential amino acids (NEAA, Thermo Fisher Scientific, Waltham, MA, USA), and 1% antifungal antibiotics (amphotericin B 0.25 μg/mL, penicillin G 100 µg/mL, streptomycin 100 μg/mL) until they reached 70–80% confluence. Then, the cells were washed with phosphate-buffered saline (PBS) twice and the culture medium was replaced with an exosome-free medium. After 24 h, the culture medium was collected for further analysis.

The EVs were isolated from the culture medium using differential centrifugation as previously described [31]. First, the culture medium in a volume of 18 mL was centrifuged at 5000× *g* for 30 min at 4 °C (SX4750A type rotor, Beckman Coulter, Allegra X-15R Centrifuge, Indianapolis, IN, USA) to remove cell debris. The resulting supernatant was passed through a 0.22 µm filter. EVs were precipitated by ultracentrifugation at 100,000× *g* (k-factor 123) for 120 min at 4 °C by Optima MAX-XP Ultracentrifuge and a TLA-55 rotor (Beckman Coulter, Indianapolis, IN, USA). The pellet was resuspended in 50 µL of 0.015% sodium cholate solution in 0.1 M PBS, pH 7.4 and stirred with a vertical rotator on a Bio RS-24 mini-rotator (Biosan SIA, Riga, Latvia) for 30 min at room temperature, followed by the ultracentrifugation step described above. The precipitate obtained as a result of the second step was dissolved in 50 µL of 0.1 M PBS, pH 7.4, and layered on 500 µL of 26% sucrose solution in PBS (ρ = 1.1082 g/mL) followed by ultracentrifugation at a rate of 120,000× *g* (k-factor 102) for 90 min at 4 °C. The resulting precipitate was dissolved in 50 µL of 0.1 M PBS, pH 7.4. The sediment was then frozen at −80 °C for subsequent proteomic analysis. Exosome isolation was performed in triplicate for each cell line.

### 4.2. Clinical Sample Description

Whole plasma samples were obtained from 11 patients with colorectal cancer (CRC) (4 women and 7 men) aged from 50 to 70 years (mean age 62 ± 6.02 years). In 9 patients, stage 3 CRC was diagnosed in (including 3, 3A, 3B), and 2 patients had stage 4 CRC. In the context of the international classification of CRC based on the TNM system, all 11 patients had metastases to the regional lymph nodes (N1, N1a, N2a), while 2 patients had distant metastases in liver and abdomen (M1). Patients with a family predisposition to CRC were not included.

Plasma samples of 20 healthy volunteers (10 men and 10 women aged 45 to 74 years; median, 59.5 years) were used in this study.

Venous blood from patients with CRC and healthy donors was collected uniformly: in identical vacuum tubes with K2 EDTA. Plasma was obtained by centrifugation of whole blood at 1300× *g* for 10 min immediately after sampling. Hemolysis was assessed by visual inspection. The resultant plasma aliquots (200 μL) were stored at −80 °C until analysis.

The resultant plasma was aliquoted, and samples were stored at −80 °C.

### 4.3. EV Isolation from Blood Plasma and Protein Extraction

EVs were obtained using the commercial Total Exosome Isolation kit (Invitrogen, Thermo Fisher Scientific, Vilnius, Lithuania) according to the manufacturer’s protocol: 6 µL of the Total Exosome Isolation reagent was added to 30 µL of whole plasma. The reagent was added to 30 μL of whole plasma, followed by incubation for 30 min at 4 °C then centrifugation at 10,000× *g* for 10 min at room temperature. The obtained precipitate was redissolved in 100 μL of 0.1 M Tris–HCl solution, (pH 8.5).

Added to the sample for further methanol–chloroform protein extraction were 400 µL of 100% methanol (J.T. Baker, Avantor, Gliwice, Poland), 100 µL of Chloroform (Sigma-Aldrich, Merc, St. Louis, MO, USA) and 300 µL of deionized water were (before each addition of a new component, the sample was thoroughly mixed by repetitive mixing). The sample was centrifuged at 14,000× *g* for 2 min at room temperature. The precipitate was redissolved in 400 µL of 100% methanol solution and then the centrifugation was repeated for 3 min. The resulting precipitate was dissolved in a buffer containing 1% sodium dodecyl sulfate (SDS) in 100 mM Tris-HCl (pH 8.5) and sonicated with a Bandelin Sonopuls ultrasonicator (Bandelin Electronic, Berlin, Germany) with a power of 30% for 30 s on ice, then centrifuged for 20 min at 14,000× *g* and 4 °C.

### 4.4. Sample Preparation for Mass Spectrometry Analysis

Sample preparation of EVs obtained from the culture medium of CRC cell lines for further targeted mass spectrometry analysis was performed as described previously [60].

Sample preparation of EVs obtained from blood plasma included protein extraction, reduction and alkylation, and hydrolytic digestion using an S-Trap micro-spin column (Protifi, Huntington, NY, USA) according to the manufacturer’s protocol. A 100 μg sample was lysed in a lysis buffer (final SDS concentration was 5% in 50 mM triethylammonium bicarbonate buffer (TEAB) (Sigma-Aldrich, St. Louis, MO, USA) (pH 8.5)) and sonicated with the Bandelin Sonopuls ultrasonicator with a power of 30% for 30 s on ice, then centrifuged for 8 min at 14,000× *g* at room temperature.

Reduction and alkylation were performed by adding tris(2-carboxyethyl)phosphine (TCEP) (Thermo Fisher Scientific, Waltham, MA, USA) and chloroacetamide (CAA) (Sigma-Aldrich, St. Louis, MO, USA) to the sample to the final concentrations of 50 mM and 80 mM, respectively. Then, the samples were incubated at 80 °C for 40 min.

A 12% solution of phosphoric acid was added to the resulting lysates, to a final concentration of 1.2%, then 5 parts of S-Trap buffer (90% methanol solution in 100 mM TEAB) were added and the sample was applied to a S-Trap column by centrifugation at 4000× *g* for 1 min at 20 °C. Then, the column was washed 4 times with the S-Trap buffer.

Trypsin solution was added to a final concentration of 0.05 µg/µL (Promega, Fitchburg, WI, USA), followed by incubation at 47 °C for 90 min. Peptide elution was performed by adding 40 μL of 0.2% formic acid to 50 mM TEAB (pH 8.5). Then, it was centrifuged at a speed of 4000× *g* for 3 min at room temperature. To elute hydrophobic peptides, 35 µL of 0.2% formic acid in 50% acetonitrile was added. In the obtained samples, the peptide concentrations were determined by the colorimetric method using a Pierce Quantitative Colorimetric Peptide Assay kit (Pierce, Rockford, IL, USA) in accordance with the manufacturer’s recommendations. Samples were dried using a vacuum concentrator SpeedVac (Thermo Scientific, Waltham, MA, USA) and resuspended in 0.1% FA solution containing SIS to final content of each SIS 40 fmol/µg of total peptides.

### 4.5. Synthesis of SIS

We expanded the SRM panel that has been developed in previous experiments on model cancerous cell lines [31]. Fibronectin 1 (FN1) gene encodes 17 distinct isoforms (according to the UniProt database). However, while most of them have a structural function in various tissues, isoform 2 is mainly expressed in the embryonic tissues and by fibroblasts in cancer patients. Due to high homogeneity of FN1 isoform sequences, there are very few tryptic peptides that are uniquely mapped onto specific isoforms. We added isoform-specific peptides (TDSTTSNYEQDQK for isoform 2 and WRPVSIPPR for isoforms 2/16), mapping to fibronectin 1 (FN1). We also added peptides DLGPPMVAR and LGVAGQWR that are mapped to DDR1 and UCHL1 proteins, which were detected in CRC cell line secretome (unpublished data). Also, peptide LTQLGTFEDHFLSLQR, which is mapped to EGFR protein and associated with different types of cancers, has been added.

For targeted mass spectrometry analysis, synthetic peptides were synthesized and used as internal standards. These standards are natural, identical peptides containing isotopically labeled amino acids (Lys or Arg 13C6,15N4) that are added as control elements in the analysis of samples. The physicochemical properties of natural peptides and their synthetic analogues do not differ; therefore, they are eluted together from the reverse-phase column, but their molecular mass differs by 8 Da (heavy Lys (K)) or 10 Da (heavy Arg (R)). By adding a known amount of synthetic peptides to the peptide mixture, the amount of the natural analogue can be calculated.

Solid-phase peptide synthesis was performed using an Overture™ Robotic Peptide Library Synthesizer (Protein Technologies, Manchester, UK), as described previously [25]. In the synthesis of isotope-labeled peptides, the isotopically labeled amino acids Fmoc-Lys-OH-13C6.15N or Fmoc-Arg-OH-13C6.15N (Cambridge Isotope Laboratories, Cambridge, MA, USA) were used instead of the usual lysine or arginine. All the peptides are listed in Supplementary Materials Table S1.

### 4.6. Quantitative Analysis of EV-Associated Proteins by Targeted Mass Spectrometry

For SRM/SIS analysis 14.5 µg of total peptide was used for each sample per LC-SRM run. Each experimental sample was analyzed in three technical replicates. Before analysis, the samples were dried in a vacuum concentrator and reconstituted in 0.1% formic acid containing SIS in an equimolar concentration of 500 fmol/µL. The final content of each SIS was 40 fmol/µg of total peptides.

Chromatographic separation was performed using an Agilent 1200 series system (Agilent Technologies, Santa Clara, CA, USA) connected to a TSQ Quantiva triple-quadrupole mass analyzer (Thermo Scientific, Waltham, MA, USA). A sample was separated using an analytical column ZORBAX SB-C18 (150 × 0.5 mm, 5 μm particle diameter) (Agilent Technologies, Santa Clara, CA, USA) in a gradient of acetonitrile with a flow rate of 20 μL/min. First, the column was equilibrated with 5% solution B (80% acetonitrile in 0.1% formic acid) and 95% solution A (0.1% formic acid) for 5 min. Then, the concentration of solution B was linearly increased to 50% for 30 min, after which the concentration of solution B was increased to 99% in 1 min and the column was washed with 99% solution B for 5 min. Then, the concentration was returned to the initial conditions for 1 min, in which the column was balanced for 9 min. Mass spectrometry analysis was performed in the dynamic selected-reaction monitoring (dSRM) mode using the following settings of the MS detector: the capillary voltage was 4000 V, the velocity of the drying gas (nitrogen) was 7 L/min, the velocity of the axillary gas (nitrogen) was 5 L/min, the capillary temperature was 350 °C, the isolation window for the first and third quadrupole was 0.7 Da, the scan cycle time was 1.2 s, and the collision gas (argon) pressure in the second quadrupole was set at 1.5 mTorr. The retention time window on the reverse phase column was 2.2 min for each precursor ion. The transition and normalized collision energy (V) lists are presented in Supplementary Table S1.

The results were analyzed and plotted using Skyline MacCoss Lab Software (version 4.1.0) to compare chromatographic profiles of the endogenous peptide and the corresponding SIS standard. The peak area ratio for the endogenous peptide and the corresponding SIS standard were automatically calculated in Skyline. To determine the amount of protein, the ratio calculated in Skyline was multiplied by the known content of each SIS standard. The measurement of each EV-associated protein was taken as the mean value of the content calculated from the results of MRM analysis in triplicate for a plasma sample after tryptic digestion, performed in a single replicate. The target protein content was expressed in fmol/μg total protein, and then converted to molar concentration in nmol/L (nM).

### 4.7. Statistical and Bioinformatic Analysis

An analysis to determine the enrichment of EV-associated proteins was performed using the Enrichr module of the GSEApy bioinformatics tool (v. 1.0.4) across categories of the DisGeNET, GO_Biological process_2021, GO_Cellular Components_2021, and GO_Molecular Function_2021 libraries; the *p*-value cutoff was <0.05. The three most significant categories were visualized.

To investigate the association of expression levels and patient survival, we used the UALCAN online platform (http://ualcan.path.uab.edu (accessed on 27 April 2023)) and data on transcript expression levels for LC patients obtained from the Cancer Genome Atlas (TCGA).

Using Python script, the results of SRM analysis in HPL-derived EV samples were analyzed with a distance-based K-means algorithm, and elbow method visualization was performed. SRM measurements for EVs and WhL derived from CRC cell lines were normalized using the MinMaxScaler function from the scikit-learnlibrary.

## 5. Conclusions

Using the multiplex mass spectrometry method, we obtained a “snapshot” of the content of vesicle-associated proteins, relevant for CRC biology and disease prognosis. The proteomic signature of 28 components allowed us to distinguish the EV and WhL samples derived from the model CRC cell lines Caco2, HT29, and HCT116. The proteomic signature of 10 components distinguished HPL/CRC-derived EV samples from healthy control. Thus, we formed a panel of EV proteins associated with CRC. The panels for targeted mass spectrometry analysis could be easily expanded with new protein analytes, which provide a proteomic signature that most effectively distinguishes CRC patients from healthy controls. The simultaneous analysis of proteomic composition CRC-associated EV is relevant to the field of liquid biopsy.

## Figures and Tables

**Table 1:** Patient and healthy volunteer characteristics. CRC—colorectal cancer, HVs—healthy volunteers.

	CRC	HVs
Total	11	20
Age	50–70	45–74
Male	7	10
Female	4	10
Stage 3, 3A	4	-
Stage 3B, 3C	5	-
Stage 4	2	-

## Data Availability

The targeted mass spectrometry data have been uploaded to the PASSEL repository (dataset PASS04826).

## Supplementary Materials

The following supporting information can be downloaded at https://www.mdpi.com/article/10.3390/molecules28104227/s1. Figure S1: Correlations between measurements of CD82, EPS15, FN1, HSPG2, MFGE8, PDCD6IP level of content for 2 unique peptides and distribution of the coefficient of variation (CV) in cell line-derived EV samples. Figure S2: Abundance of 34 peptides uniquely mapped onto 28 EV-associated proteins. Figure S3: Correlations between measurements of FN1 levels of content for 2 unique peptides and distribution of the coefficient of variation (CV) in HPL-derived EV samples. Figure S4: Trace of SRM transitions for 12 peptides detected in blood plasma for EV-associated proteins. Figure S5. Abundance of 3 convenient markers of exosome HSPA8, CD9, and CD63 in HPL-derived EV samples. Table S1: SRM measurement of media-derived EV, HPL-derived EV, peptides, and proteins. Table S2: Clinical sample characterization.
