# Supplementary material for: Proteomic Signature of Extracellular Vesicles Associated with Colorectal Cancer
Source: Molecules. 2023 May 22;28(10):4227. doi: 10.3390/molecules28104227 (PMC10222552; doi:10.3390/molecules28104227)
Supplement: Supplementary file 1 [file molecules-28-04227-s001.zip › Supplementary materials_v2.pdf]

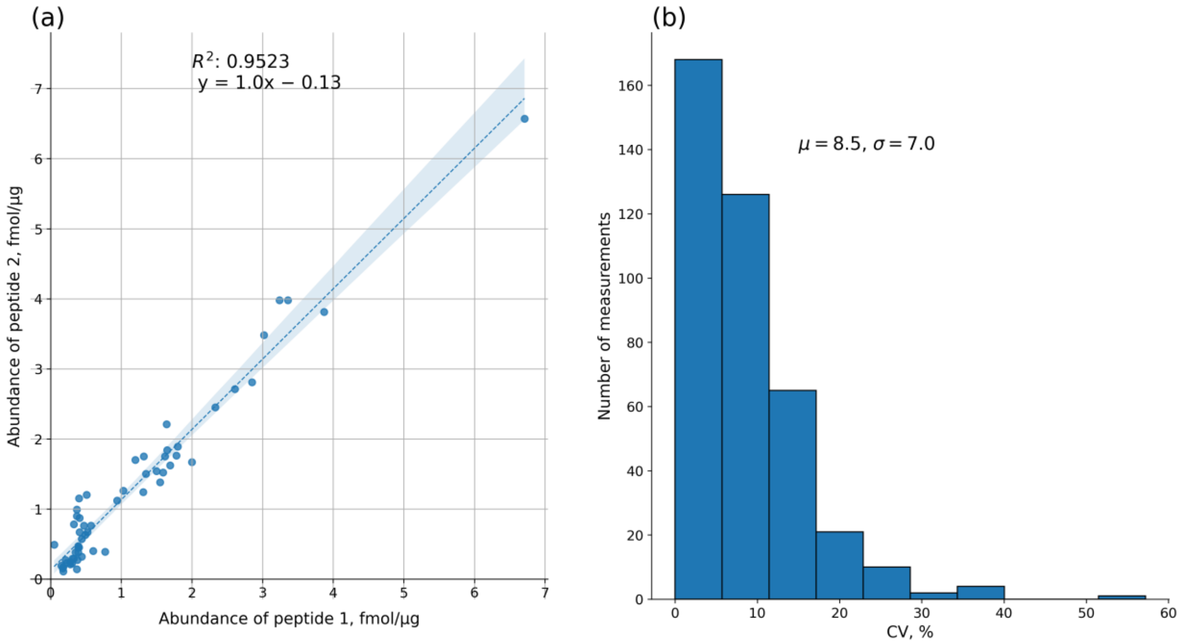


Figure S1. (a) Correlation of content for 2 unique peptides mapped onto CD82, EPS15, FN1, HSPG2, MFGE8, PDCD6IP, and TSG101 proteins measured in cultural media-derived EV samples;(b) distribution of the coefficient of variation (CV, %) for all measurements performed in tree technical replicates in media-derived EV samples.

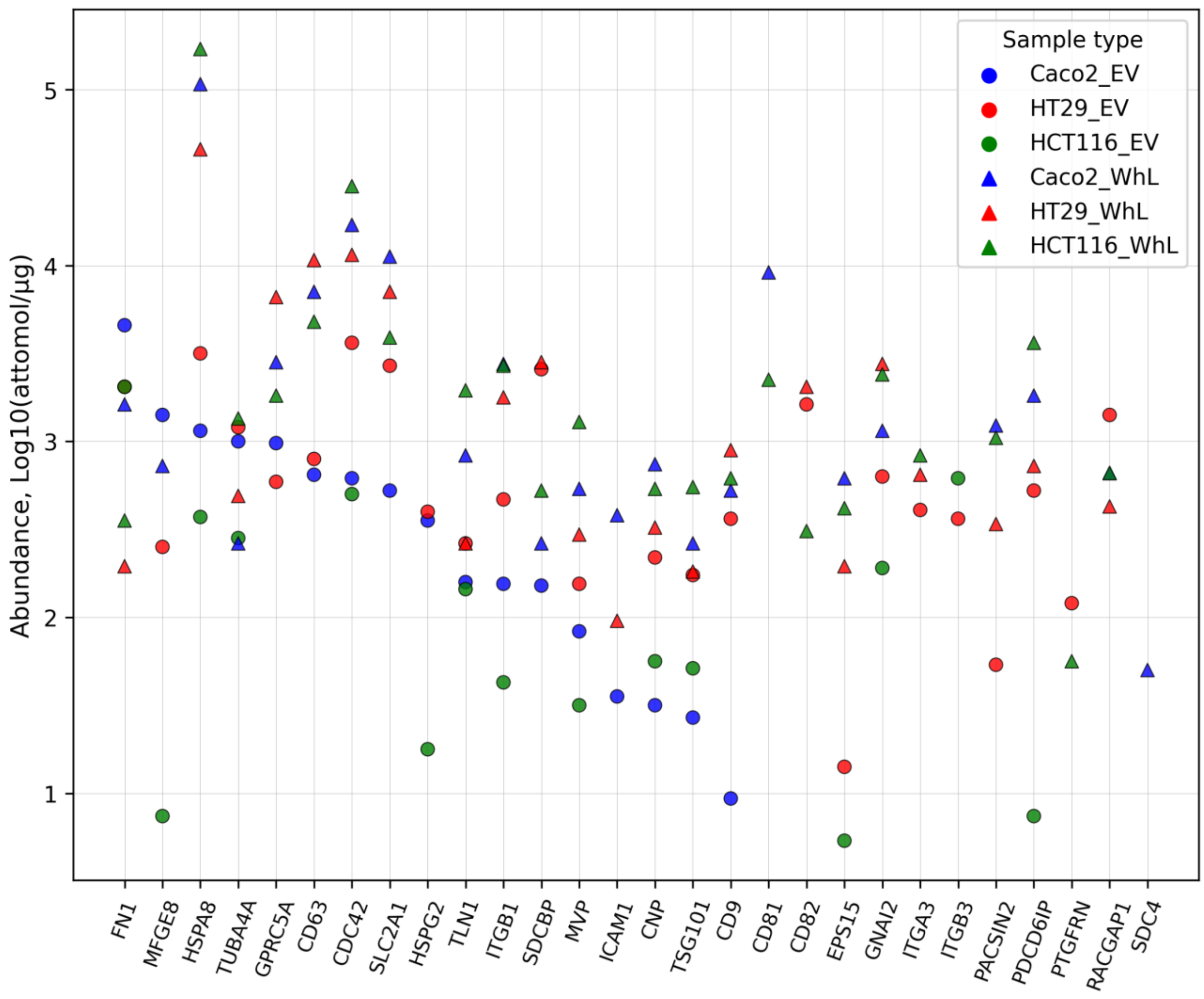


Figure S2. Abundance of 34 peptides uniquely mapped onto 28 EV-associated proteins, which were measured in the extracellular vesicles (EV) and whole cell lysate (WhL) samples derived from CRC HT29, HCT-116, and CaCo-2 cell lines; Y-axis is absolute protein content in amol/µg of total peptide, log10-transformed;

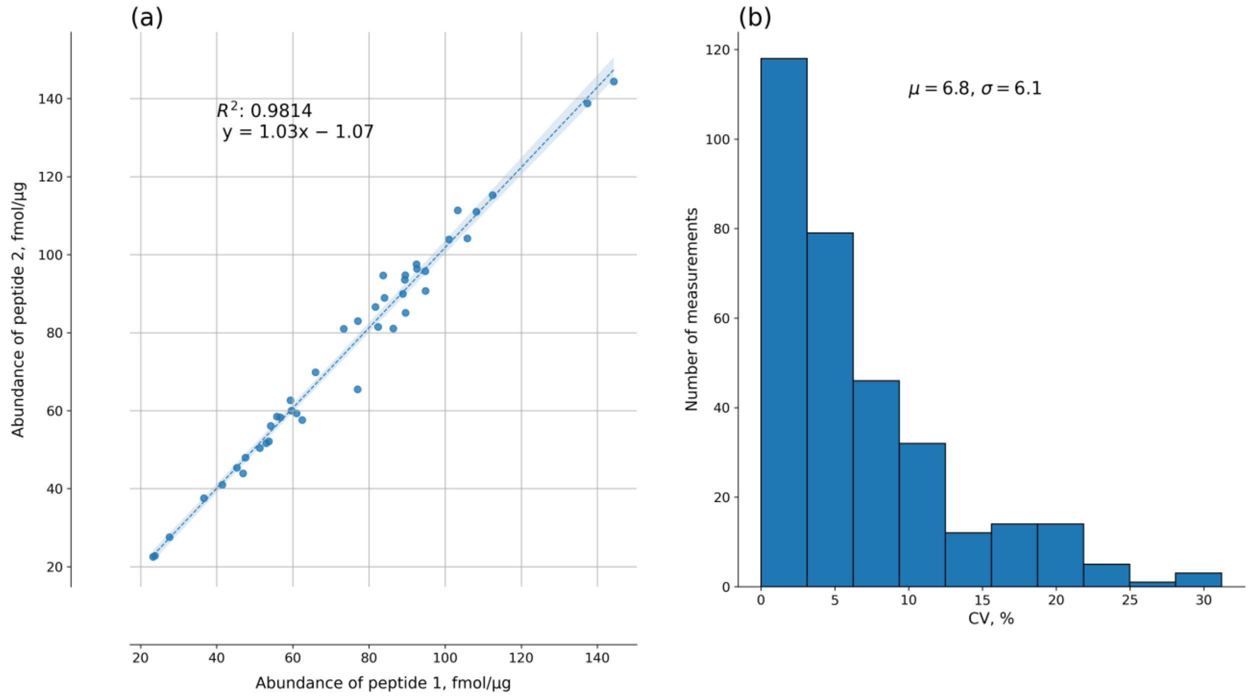


Figure S3. (a) Correlation of content for 2 unique peptides mapped onto FN1 protein measured in HPL-derived EV samples; (b) distribution of the coefficient of variation (CV, %) for all measurements performed in tree technical replicates in HPL-derived EV samples.

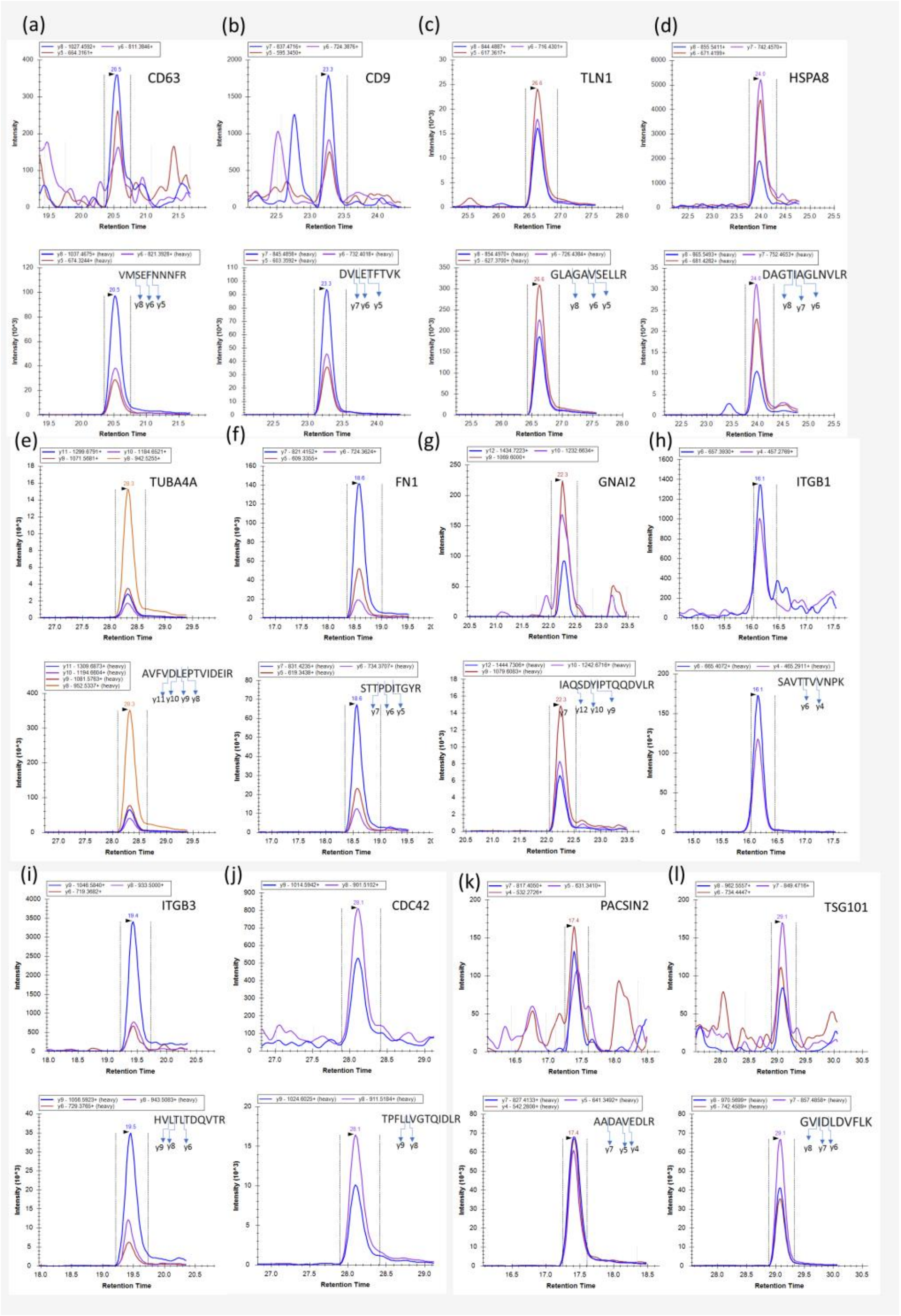


Figure S4. Trace of SRM transitions for native (upper panel) and stable isotope-labeled peptide standards (SISs) (lower panel) detected in blood plasma for EV-associated proteins: (a) CD63 (peptide VMSEFNNNFR), (b) CD9 (peptide DVLETFTVK), (c) TLN1 (peptide GLAGAVSELLR), (d) HSPA8 (peptide DAGTIAGLNVLR), (e) TUBA4A (peptide AVFVDLEPTVIDEIR) (f) FN1 (peptide STTPDITGYR) (g) GNAI2 (peptide IAQSDYIPTQQDVLR) (h) ITGB1 (peptide

SAVTTVVNPK), (i) ITGB3 (peptide HVLTLTDQVTR), (j) CDC42 (peptide TPFLLVGTQIDLR), (k) PACSIN2 (peptide AADAVEDLR) and (l) TSG101 (peptide GVIDLDVFLK)

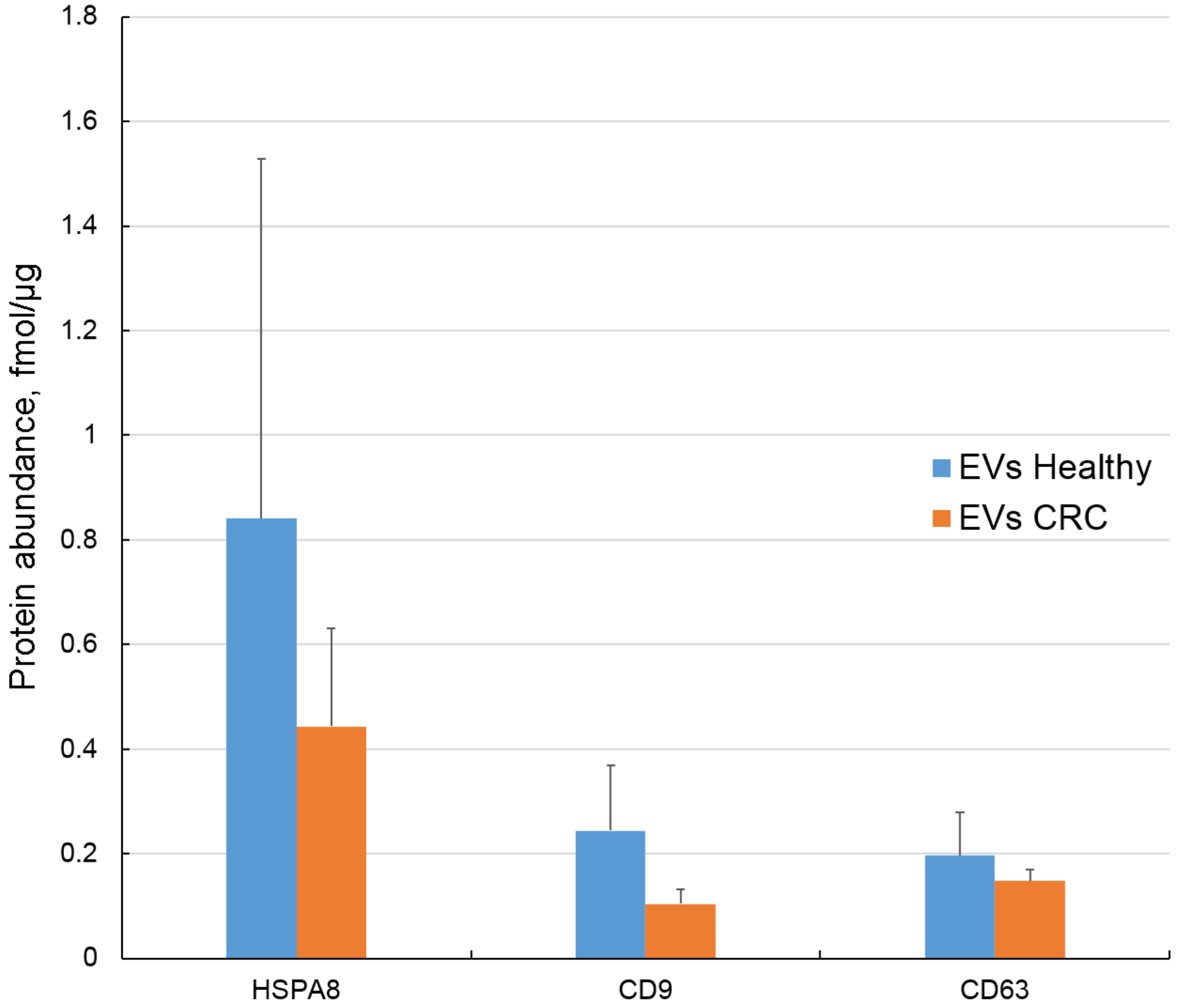


Figure S5. Abundance of 3 convenient markers of exosome HSPA8, CD9, and CD63 in HPL-derived EV samples from patients with CRC and healthy donors.
